# Effect of Graphene Oxide and Nanosilica Modifications on Electrospun Core-Shell PVA–PEG–SiO_2_@PVA–GO Fiber Mats

**DOI:** 10.3390/nano12060998

**Published:** 2022-03-18

**Authors:** Yuliya Kan, Julia V. Bondareva, Eugene S. Statnik, Julijana Cvjetinovic, Svetlana Lipovskikh, Arkady S. Abdurashitov, Maria A. Kirsanova, Gleb B. Sukhorukhov, Stanislav A. Evlashin, Alexey I. Salimon, Alexander M. Korsunsky

**Affiliations:** 1 Center for Energy Science and Technology, Skolkovo Institute of Science and Technology, 3 Nobel Street, 143026 Moscow, Russia; eugene.statnik@skoltech.ru (E.S.S.); s.lipovskikh@skoltech.ru (S.L.); maria.kirsanova@skoltech.ru (M.A.K.); a.salimon@skoltech.ru (A.I.S.); 2 Center for Materials Technologies, Skolkovo Institute of Science and Technology, Bolshoy Boulevard 30, bld. 1, 121205 Moscow, Russia; j.bondareva@skoltech.ru (J.V.B.); s.evlashin@skoltech.ru (S.A.E.); 3 Center for Photonic Science and Engineering, Skolkovo Institute of Science and Technology, 3 Nobel Street, 143026 Moscow, Russia; julijana.cvjetinovic@skoltech.ru; 4 Center for Neurobiology and Brain Restoration, Skolkovo Institute of Science and Technology, 3 Nobel Street, 143026 Moscow, Russia; a.abdurashitov@skoltech.ru (A.S.A.); g.sukhorukov@skoltech.ru (G.B.S.); 5 School of Engineering and Materials Science, Queen Mary University of London, Mile End Road, London E1 4NS, UK; 6 Multi-Beam Laboratory for Engineering Microscopy (MBLEM), Department of Engineering Science, University of Oxford, Parks Road, Oxford OX1 3PJ, UK; alexander.korsunsky@eng.ox.ac.uk

**Keywords:** electrospinning, core-shell nanofibers, drug delivery, graphene oxide, fiber modification, silica

## Abstract

Electrospinning is a well-established method for the fabrication of polymer biomaterials, including those with core-shell nanofibers. The variability of structures presents a great range of opportunities in tissue engineering and drug delivery by incorporating biologically active molecules such as drugs, proteins, and growth factors and subsequent control of their release into the target microenvironment to achieve therapeutic effect. The object of study is non-woven core-shell PVA–PEG–SiO_2_@PVA–GO fiber mats assembled by the technology of coaxial electrospinning. The task of the core-shell fiber development was set to regulate the degradation process under external factors. The dual structure was modified with silica nanoparticles and graphene oxide to ensure the fiber integrity and stability. The influence of the nano additives and crosslinking conditions for the composite was investigated as a function of fiber diameter, hydrolysis, and mechanical properties. Tensile mechanical tests and water degradation tests were used to reveal the fracture and dissolution behavior of the fiber mats and bundles. The obtained fibers were visualized by confocal fluorescence microscopy to confirm the continuous core-shell structure and encapsulation feasibility for biologically active components, selectively in the fiber core and shell. The results provide a firm basis to draw the conclusion that electrospun core-shell fiber mats have tremendous potential for biomedical applications as drug carriers, photocatalysts, and wound dressings.

## 1. Introduction

Electrospinning, introduced and developed in the 1980s [1], remains the subject of attention for researchers as a versatile technology for producing fibers with a diverse range of diameters, morphologies, and functions. Electrospun fiber mats have found application as filters [2], membranes, substrates for tissue engineering and growth [3,4,5,6], wound dressings [7,8,9], and photocatalysts for water defluoridation and cleansing [10,11]. The properties of the fibers depend on various polymer frameworks, which differ in their biocompatibility, biodegradability, hydrophobicity, and water solubility.

Electrospun polymers have interesting applications in the biomedical field, for instance, the hydrophilic polyvinyl alcohol (PVA) polymer provides good miscibility with water-soluble drugs. In our previous study, we already discussed the PVA-based fibrous biodegradable materials for tissue engineering [12]. The limitation of electrospun materials based on PVA is a relatively low mechanical strength that prevents their use in structural applications. However, degradability becomes advantageous when the designed application concerns the abrupt or gradual release of substance-loaded fibers into the environment, where the structure’s morphology affects the rate of degradation corresponding to the drug release capability of a fiber mat. According to studies [13,14,15], polymer biodegradability holds promise for controlled delivery of encapsulated drugs, with the characteristic release time related to the polymer degradation rate. Thus, considering the high repeatability of PVA solutions and mentioned limitations, the development of the existing PVA biomaterials aims to create a resistant structure for the gradual drug release. One way to overcome the limitations is to create a fiber with a core-shell structure. This technology facilitates dual modification of the core and the shell to improve the functions of fibers. In the biomedical field, the bioactive molecules’ inclusion in the core layer is a promising trend, whereas the shield layer acts as a barrier and determines the release of biomolecules. Suggested modification of shell and core materials is required to achieve the sustainable release rate and ensure proper adhesion between the core and shell of the fibers.

Another issue of electrospinning is low bulkiness, such that a sufficient thickness of mat is required to withstand the stress and drug loading strategies. Considering the potential in the biomedical field, after the fiber was formed, the bulk volume of the fiber mat could be successfully optimized by molding technology. There is growing interest in the microsystems patterned with mold to support local drug administration to the tumor area [16], with a high drug load capacity introduced [17].

Nanomaterials such as graphene oxide and nanosilica are prospective and widespread compounds for modifying the composite. Increasing the mechanical strength of fiber is a driving force for a composite consisting of the polymer network with embedded nanomaterials. Graphene oxide (GO) can be used to improve the mechanical properties of non-woven electrospun fiber mats, and reinforcement of PVA-based composites with graphene oxide has already been proposed in the following studies [18,19,20]. The high surface area and high miscibility of the composite obtained from GO is considered effective for an absorbent with a high level of interaction in aqueous conditions [21]. The hydrophilicity of GO is suitable for water-soluble PVA and polyethylene glycol (PEG) to form strong hydrogen bonds within the composite, providing mechanical stability to the core-shell fiber. Additionally, the GO and GO derived nanomaterials have the adjustable fluorescence properties that are applicable for bioimaging of tumor and other biomedical therapies with the encapsulation of drug molecules [22,23]. GO toxicity remains controversial, and working concentration is under investigation. In some studies, the lowest concentration reaches 1.56 µg/mL for the viable cell response of epithelial lung cells [24]. According to the study [25], the upper limit of GO concentration, 25 µg/mL in distilled water, caused a 50% decrease in the human skin fibroblast cells’ viability in the assay after 24 h. Hence, the operational concentration should be carefully considered to create biocompatible core-shell fibers.

In regard to the targeted drug delivery, a polymer matrix could be a carrier, but if the drug solubility does not match the polymer, then an additional component is required. Silica can be considered as a co-delivery container for drug loading due to its high surface area. For instance, the water-soluble (Rhodamine B) and hydrophobic dyes (Fluorescein) could be placed in silica and the polymer matrix separately and serve as a drug carrier system [26,27]. PEG was suggested as a stabilizing agent to build core PVA–PEG–SiO_2_ hybrid and control aggregation of nanoparticles [28]. The modification of PVA with silica precursor tetraethyl orthosilicate (TEOS) caused the crosslinking mechanism through chemical bonds Si−O−C−O−Si [29]. It affects the integrity of the composite and expands the functionality of non-woven materials with a variety of synthesized fibers by alternating the rheology of spinning solutions.

In this work, the following concept of the coaxial electrospinning was used to build the non-woven fiber mats consisting of core-shell fibers PVA–PEG–SiO_2_@PVA–GO. The dual modification with silica nanoparticles and graphene oxide is expected to provide the local stability of the core-shell fiber composite. PVA–GO is a shell of fiber, where graphene oxide improves the mechanical properties and extends the elasticity of composite relative to the core structure. PVA–PEG–SiO_2_ composite is a core of the fiber, where silica nanoparticles can enhance the structure of the polymer matrix and enter into host–guest interactions for targeted delivery of drug molecules. The core-shell fiber mat with pattern was aimed at exploring the functionality in the scope of drug delivery applications.

## 2. Materials and Methods

### 2.1. Chemical Reagents

PVA powder with the molecular weight (MW) 72,000 g/mol and with the degree of hydrolysis 85–89% and PEG 8000 BioChemica with MW 7000–9000 g/mol were supplied from AppliChem GmbH, Darmstadt, Germany. Phosphate buffered saline (PBS), fluorescent dyes fluorescein isothiocyanate (FITC), and Rhodamine B were purchased from Sigma-Aldrich, St. Louis, MO, USA. Methanol (≥99.5%) was supplied from Chimmed, Moscow, Russia.

### 2.2. Synthesis of Graphene Oxide and Silica

GO was obtained by oxidation of graphite flakes Timrex 15 (Timcal Ltd., Bodio, Switzerland) using a conventional Hummers’ method [30] and described in our previous studies [31,32,33]. Next, 69 mL of sulfuric acid (H_2_SO_4_) was poured into a 500 mL glass beaker, 3 g of graphite was successively added under stirring and cooling conditions, then 1.5 g of sodium nitrate (NaNO_3_) and 9 g of potassium permanganate (KMnO_4_) were added. The resulting dark-brownish mixture was left for 2 h with constant stirring. Then, 300 mL of water was added in portions to the resulting mixture, after which, 30 mL hydrogen peroxide (H_2_O_2_) was slowly added dropwise and stirred for 10 min to eliminate excess unreacted KMnO_4_. After adding H_2_O_2_ to the mixture, the color turned to light yellow. The mixture was left overnight. Then, a yellow precipitate was decanted from the solution. The residue was washed with water five times, centrifuged at 5500 rpm on OPN-16 Benchtop High-Speed Centrifuge (Labtech, Moscow, Russia).

Silica nanoparticles were obtained from the P-type silicon wafer by hydrothermal synthesis. Silicon was added to 60 mL aqueous ammonia medium with a concentration of 0.53 M into Teflon-lined sealed stainless-steel autoclaves and maintained at 180 °C for 24 h under autogenous pressure. The ready-to-use suspensions of silica oxide nanoparticles (NPs) with a concentration of 0.8 g/l and graphene oxide with a concentration of 30 g/L were obtained by the protocol of fabrication mentioned in the reference studies [32,33]. The concentrations of as-received NPs of silica and GO solutions were measured with DTG 60 (Shimadzu, Kyoto, Japan).

### 2.3. Polymer Solutions Preparation

To assess the effect of the additive components on the fiber morphology, the following lists of the solutions were prepared (Table 1). At 80 °C, 10 wt.% PVA solution was made at the magnetic stirrer by dissolving the polymer in the distilled water for 2 h and used as a base solution.

As the core solution, the PVA and PEG were mixed in the ratio 70:30 and homogenized at 80 °C in the magnetic stirrer Hei-Standard (d = 145 mm, Heidolph Instruments GmbH & Co. KG, Schwabach, Germany) for 2 h with a mass ratio of polymer 12 wt.%. Then, 0.005 wt.% of silica suspension was dropped to the polymeric solution and sonicated in the ultrasound (US) bath Elmasonic S10H (Elma Schmidbauer GmbH, Singen, Germany) for 10 min. The solutions were degassed and cooled down to room temperature. The final concentration of NPs of silica was 0.049 mg/mL in the whole PVA–PEG–SiO_2_ solution. The prepared PVA-PEG solutions and PVA–PEG–SiO_2_ were studied to trace the effect of nanosilica.

For the shell solution, the graphene oxide was mixed with the base polymer solution of the PVA 10 wt.% prepared at 80 °C with a stirring rate of 250 rpm for 2 h. As-received graphene oxide solution was sonicated in the Elmasonic S10H (Elma Schmidbauer GmbH, Singen, Germany) for 10 min and added to the PVA solution with the loading of 5 and 10 wt.%. The final concentration of GO was 1.21 mg/mL and 2.42 mg/mL for PVA–GO 5% and PVA–GO 10%, respectively.

### 2.4. Fiber Mat Preparation via Electrospinning

**Figure 1:**
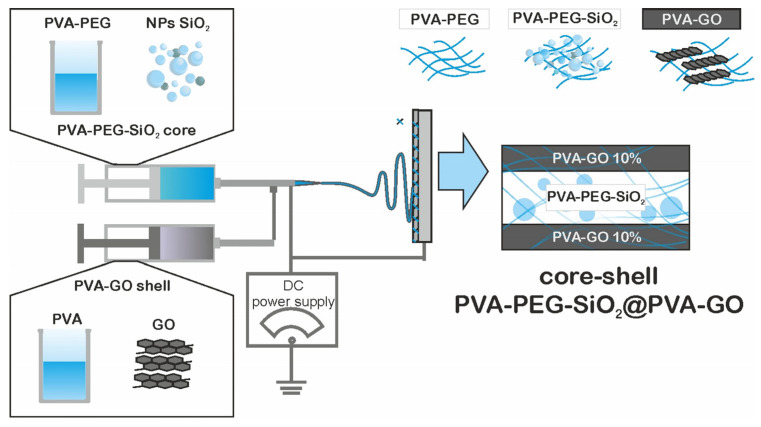
The scheme of the fiber formation.

The laboratory setup of electrospinning was constructed by the FabLab and Machine Shop Shared Facility (FabLab) of Skoltech, Moscow, Russia. The setup was switched to the high-voltage direct current power supply 21 kV; the detailed description of the setup was given in our previous study [12]. After the homogenization and degassing, the prepared core and shell solutions were placed in the syringes equipped with a coaxial nozzle (Figure 1). The supply of solution was carried out with help of the pump with a flow rate of 1 mL/h. The fiber collectors were the patterned and as-received alumina plates. The fixed collector was placed at a distance of 13 cm from the solution supply.

The fiber collector is an important part of the setup in terms of fiber manufacturing performance and the relief and morphology of the composite. The harvest of fibers depends on the induced electric field between the syringe needle and the conductive collector. During spinning, the fibrous mat reduces the conductivity of the collector and leads to a deficit in the electromagnetic field. The performance of the method can be investigated by the collector adjusting to increase the thickness and volume of the obtained material.

The modification of the fiber collector was conducted with the laser ablation method [34]. The pattern of pits was engraved in the aluminum plate with the following parameters: laser wavelength 532 nm and 20 pulses per pit, where each pulse is 50 μJ.

After the synthesis for 1 h, the fiber mat was delaminated from the collector and placed in the Petri dish to crosslink with methanol. The specimens were stored in methanol for 12 h and then left in the open air to allow the methanol to evaporate.

### 2.5. Spinning Solutions Characterization

Viscosity measurements of spinning solutions were conducted at room temperature using EMS-1000 (Kyoto Electronics Manufacturing Co., Ltd., Tokyo, Japan). The aluminum sphere (d = 2 mm) was rotated by the induced electromagnetic field with the rate of 1000 rpm during 5 s for the PVA–GO 5% and with 500 rpm during 5 s for PVA 10%, 500 rpm during 10 s for PVA–GO 10%, 500 rpm during 5 s at PVA-PEG, and 500 rpm for 30 s at PVA–PEG–SiO_2_. The volume electrical conductivity of the spinning solutions was measured with the conductometer ST10C-B (Ohaus, Nänikon, Switzerland) for 10 mL of sample volume.

### 2.6. Morphology Characterization of Fiber Mats

The morphology of the obtained fibrous mat was studied with the optical microscope Altami MET 6T (Altami Ltd., St. Petersburg, Russia), scanning electron microscope (SEM) Tescan Vega3 (TESCAN ORSAY HOLDING, Brno, Czech Republic), and FEI Helios G4 Plasma FIB Uxe (Thermo Fisher Scientific, Landsmeer, Netherlands). The samples were preliminary gold-sputtered for 10 s with 25 mA at the Quorum Q150R ES coater (East Sussex, UK) to reduce the charging. At least 100 measurements per sample were collected to build the size distribution histograms and calculate the mean diameter.

For transmission electron microscopy (TEM) observations, the PVA–PEG–SiO_2_ core fibers were collected for 5 min on top of the 300 Mesh Copper TEM support grid with lacey carbon films (Agar Scientific Ltd., Stansted, UK). Bright-field (BF) TEM and high-angle annular dark-field scanning transmission electron microscopy (HAADF-STEM) images, energy-dispersive X-ray (EDX) spectra, and compositional maps were taken on an aberration-corrected Titan Themis Z transmission electron microscope (Thermo Fisher Scientific, Landsmeer, Netherlands) equipped with a Super-X detection system and operated at 120 kV.

The core-shell structure was monitored with the fluorescence confocal laser scanning microscope LSM 800 with Airyscan (Carl Zeiss Microscopy GmbH, Jena, Germany) to claim the presence of bilayer structure. The dyes FITC and Rhodamine B were added to the core and shell solutions with a 0.01 mg/mL concentration, respectively. The samples of fibers were synthesized on top of the glass slide for 3 min to obtain the discrete fiber network.

### 2.7. Wettability of Specimens

Fiber mats collected on a glass slide for 1 h of spinning were used for the wettability measurements. Five measurements per sample were conducted to determine the contact angle with the Krüss MBL2000 microscope (KRÜSS Optronic GmbH, Hamburg, Germany) and analyzed with the integrated software.

### 2.8. Spectroscopic Characterization of Solutions and Fibers

The samples of the as-received electrospun mats and their components were studied to monitor the peaks attributed to the components and specific ‘fingerprints’ with DXR3xi Raman imaging microscope (Thermo Fisher Scientific, Waltham, MA, USA). Raman spectroscopy was conducted with the laser source 532 nm, where GO and PVA–GO samples were studied at the laser power of 3 mW and 5 mW, respectively. PVA–PEG–SiO_2_ and freeze-dried NPs of SiO_2_ solution were studied at 6 mW and 4.2 mW of laser power, respectively. Raman spectra of samples were captured in the range of 200–3200 cm^–1^.

The PVA–GO composites and the crosslinked fiber films of PVA–PEG–SiO_2_@PVA–GO were placed in 0.01 M of the PBS with pH 7.4, at 25 °C for 24 h in the orbital mixer. After 24 h of stirring, the supernatants of solutions were placed in a 96-well plate (Corning 96-well Clear Flat Bottom UV-Transparent Microplate), and fluorescence spectra were recorded with an Infinite M Nano+ (Tecan Trading AG, Männedorf, Switzerland) dual-mode microplate reader. The fluorescence from the samples was excited at 330 nm, and their emission was observed in the range of 350−800 nm.

### 2.9. Mechanical Testing with DEBEN MT 200 N

The prepared specimens 25 mm × 14 mm with two cuts (2 mm per notch) were tested by Deben Microtest 200 N Tensile Stage (Deben UK Ltd., Woolpit, UK) in tension mode with permanent speed 1.0 mm/min inside the chamber of Tescan Vega3 SEM (TESCAN ORSAY HOLDING, Brno, Czech Republic). The tension was parallel synchronized with SEM images’ acquisition with the following settings: Back-scattered Electron (BSE) detector, resolution 1024 × 1024 pixels, 8 bit, scan speed 3.2 μm per pixel, and field of view 1 mm x 1 mm. Then, images were processed by the robust Digital Image Correlation (DIC) method to obtain true material deformations. The DIC technique was realized by open-source Matlab-based software Ncorr v.1.2 (GitHub, Inc., San Francisco, CA, USA) [35]. Two characteristics were determined, namely, elastic modulus and ultimate strength.

## 3. Results and Discussions

### 3.1. Spinning Solutions Characterization

The measurements were carried out at the same room temperature as the electrospinning (Table 2). The impact of the dual polymer mixture was considered. The viscosity of PVA 10 wt.% base solution shows 1058.9 mPa∙s at a shear rate of 3.1224 s^–1^. The smaller contribution of PVA (8.4 wt.%) for PVA-PEG against the 10 wt.% of the base shows the diminishing viscosity for PVA-PEG solution to 339.8 mPa⋅s. Moreover, PEG with the ratio of 3.6 wt.% did not dramatically change the viscosity behavior of the compositions.

GO and SiO_2_ are expected to increase the viscosity from the mentioned references, showing the thickening effect of nanosilica obtained from TEOS precursors [29]. The final fraction of silica 0.005 wt.% is negligible to provide a significant contribution. The liquid part of as-received silica solution diluted PVA-PEG and caused the drop in the viscosity and changed shear behavior of the PVA–PEG–SiO_2_ solution.

The modification of the PVA base polymer solution with as-received aqueous GO solution showed the decrease in viscosity, with more GO fraction. Since the final concentration of GO is 0.242 wt.% and 0.120 wt.% for PVA–GO 10% and PVA–GO 5%, respectively, the remaining liquid part dissolves the initial polymer compound, resulting in the decreased viscosity for PVA–GO.

The liquid part of the silica solution diluted the core solution PVA–PEG–SiO_2_, where the conductivity of PVA-PEG was increased to 1332 μS∙cm^–1^. PVA–GO composite showed the degrading conductivity correlated with polymer dissolution with as-prepared GO slurry. According to the evidence of non-conductive properties of GO [36,37], the final concentration of GO in solution did not dramatically affect the conductivity.

The measurements will be used to optimize the fabrication of the core-shell fiber where the core includes a composite of PVA-PEG or PVA–PEG–SiO_2_, and the shell consists of PVA–GO 10%.

### 3.2. Electrospun Fiber Mat Characterization

#### 3.2.1. Morphology Characterization

##### SEM Study of the As-Received Fibers

**Figure 2:**
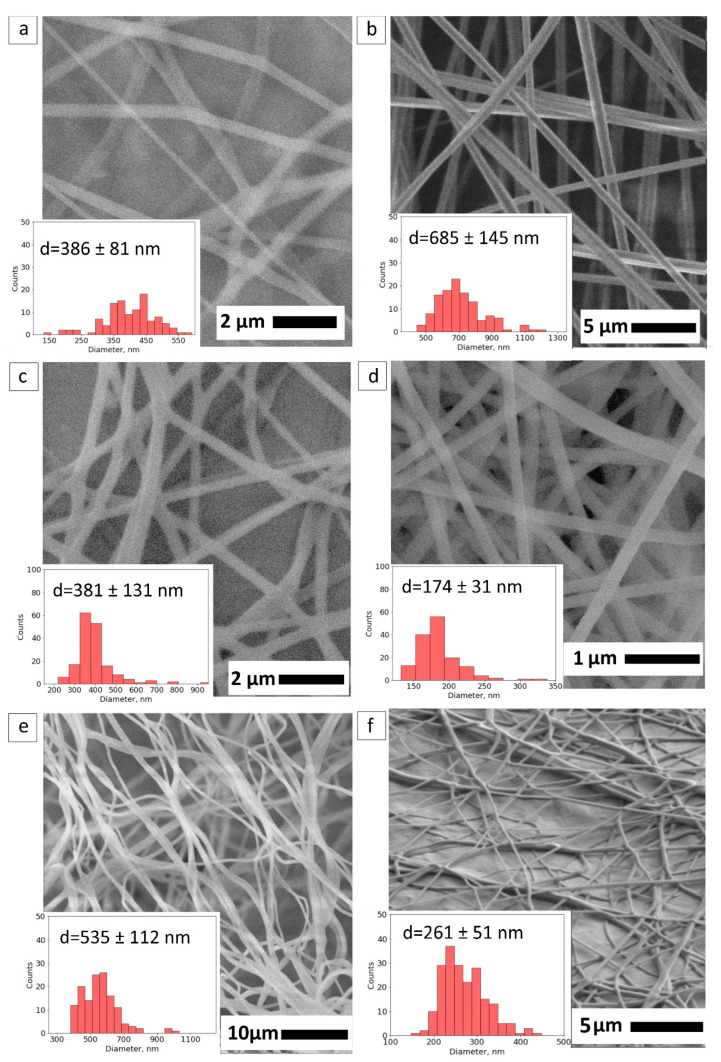
SEM imaging of fibers: (**a**) pristine PVA; (**b**) PVA-PEG core; (**c**) PVA–PEG–SiO_2_ core; (**d**) shell PVA–GO 10%; (**e**) core-shell PVA-PEG@PVA–GO); and (**f**) core-shell PVA–PEG–SiO_2_@PVA–GO.

The morphology of synthesized fiber mats was observed by SEM. The diameter of fibers from each fiber mat was calculated to monitor the effect of additives and methanol crosslinking. The as-received PVA fiber mat has a mean fiber diameter of 386 ± 81 nm in Figure 2a. In Figure 2b, PVA-PEG presents the bigger fiber size distribution with a mean fiber size of 685 ± 145 nm.

With the addition of SiO_2_, the mean size of core fiber was slightly decreased to 381 ± 131 nm (Figure 2c). The presence of nanoparticles potentially reduces the movement of polymer chains by the emerging covalent bonds between silica and the polymer matrix that result in the formation of the diminished fiber size under the electric field. In Section 3.4.1, we monitored the chemical bonds that occurred from the silica nanoparticles in the spectra of PVA–PEG–SiO_2_ fiber.

Figure 2d illustrates the effect of GO content on the PVA-based fiber. PVA–GO 10% demonstrates the decreased fiber diameter to 174 ± 31 nm compared to the pristine PVA 10% fibers. The loading of GO results in the narrowing of fiber diameters for stable electrospinning of mats with the same operational parameters.

The decreasing trend of morphology is observed for the core-shell fibers PVA-PEG@PVA–GO and PVA–PEG–SiO_2_@PVA–GO given in Figure 2e,f, respectively. At the same time, the mean diameters of the core-shell fibers of PVA-PEG@PVA–GO and PVA–PEG–SiO_2_@PVA–GO are 535 ± 112 nm and 261 ± 51 nm, respectively. The decreased fiber diameter could relate to changing of the physical properties for spinning solutions, such as viscosity and conductivity, mentioned in Table 2.

##### SEM Study of the Crosslinked Fibers

**Figure 3:**
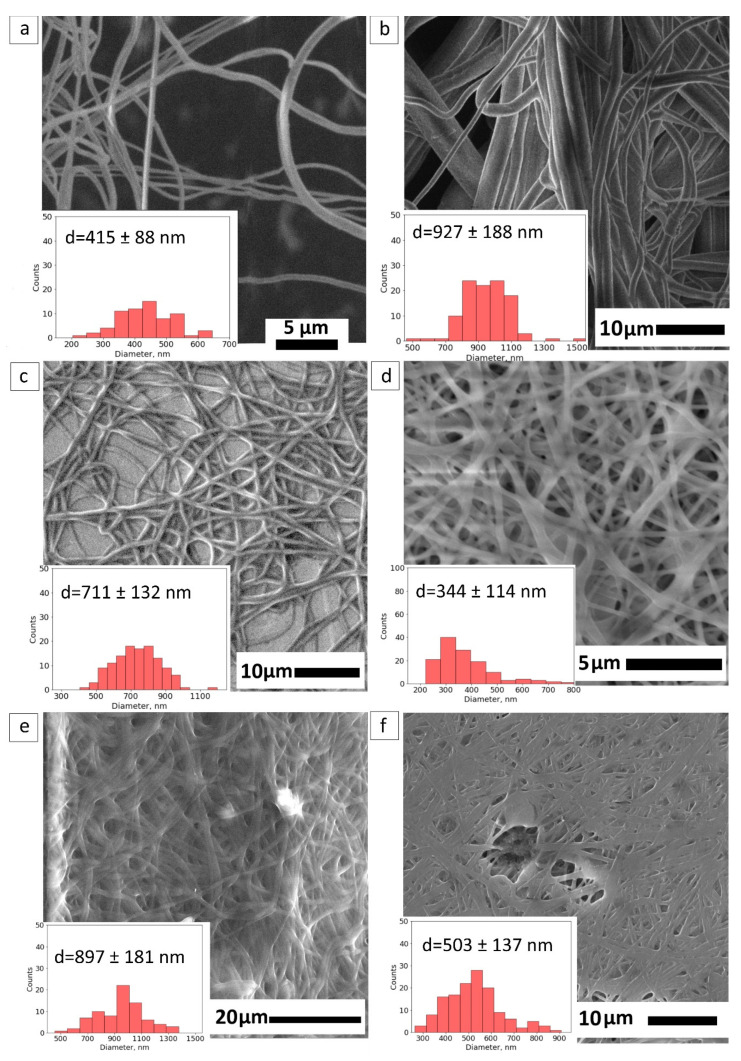
SEM imaging of crosslinked fibers with methanol: (**a**) pristine PVA; (**b**) PVA-PEG core; (**c**) PVA–PEG–SiO_2_ core; (**d**) shell PVA–GO 10%; (**e**) core-shell PVA-PEG@PVA–GO); and (**f**) core-shell PVA–PEG–SiO_2_@PVA–GO.

To increase the structural integrity of the PVA-based composite, the procedure of crosslinking with methanol was suggested. Considering the simplicity of the method mentioned in the study [38], crosslinking with methanol was chosen. Methanol evaporates in room conditions and is suitable for the robust fabrication of fiber mats. A crosslinked PVA fiber mat performs a mean diameter of 415 ± 88 nm in Figure 3a.

Figure 3b,c demonstrates the morphology of crosslinked samples starting from the core compositions: PVA-PEG, PVA–PEG–SiO_2_. The crosslinked fibers of PVA-PEG retain the fibrous structure with a wide range of sizes starting from 400 nm to 1200 nm, shown in Figure 3b. The mean size of crosslinked PVA-PEG fibers is 927 ± 188 nm. The core solution PVA–PEG–SiO_2_ provides the uniform size distribution of fibers 711 ± 132 nm in Figure 3c and prevents the swelling of the composite.

The PVA–GO-based composite as the shell is presented in Figure 3d. The mean size of fibers changes to 344 ± 114 nm after crosslinking treatment. Despite the GO’s solubility in methanol, the presence of GO was claimed in the polymer matrix with the spectroscopic methods in Section 3.4.2.

Figure 3e,f demonstrates the same trend of the diameter diminishing for the crosslinked core-shell PVA-PEG@PVA–GO against PVA–PEG–SiO_2_@PVA–GO, with the mean diameter 897 ± 181 nm and 503 ± 137 nm, respectively. The methanol vapor treatment facilitates the crosslinking of polymer chains to maintain the fiber morphology. Methanol plays the main role in stabilizing the polymer network due to the hydrogen bonds formed within the polymer chains [38].

#### 3.2.2. Molding of Obtained Fiber Mats with Microbaskets

**Figure 4:**
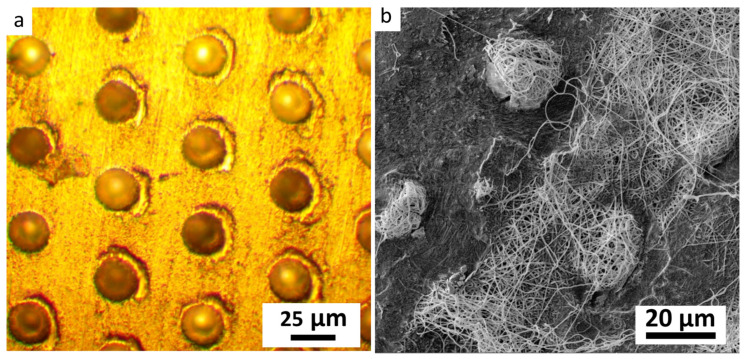
SEM images of (**a**) alumina pattern; (**b**) micro baskets of core-shell PVA–PEG–SiO_2_@PVA–GO.

The molding of fiber mats was conducted with the alumina target with the ablated pattern of pits with a diameter of 24 μm. The existing systems as microchambers [17,34] inspired the application of the lithography techniques to obtain the fiber micro relief for the higher thickness of the obtained films. The optical image of the modified fiber collector was provided in Figure 4a. After the spinning of solutions and delamination, the core-shell fiber mat repeats the relief of the collector through the microbaskets’ formation. The fibrous structure was retained and reminiscent of the basket in Figure 4b. This stage of fabrication is under development to increase the bulkiness of the synthesized material and expand the potential drug loading volume for the system.

#### 3.2.3. Silica Distribution Analysis by TEM in Core Fiber

**Figure 5:**
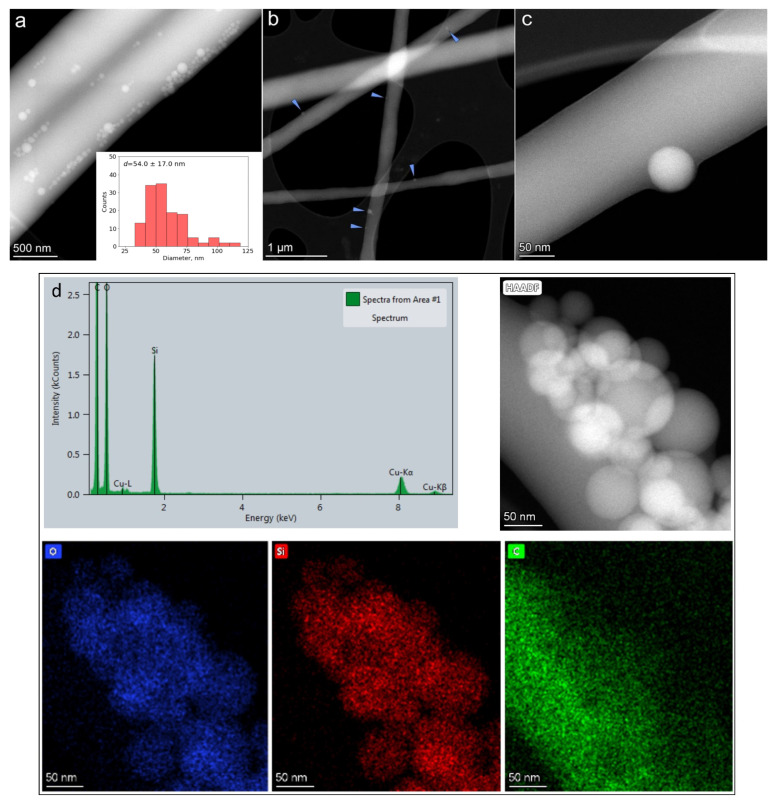
HAADF-STEM images of: (**a**) PVA–PEG–SiO_2_ with PVA:PEG (90:10); (**b**,**c**) PVA–PEG–SiO_2_ with PVA:PEG (70:30). The histogram of silica particle size distribution is shown as an inset. Blue arrowheads indicate the location of individual silica particles on the fiber; (**d**) EDX analysis of PVA–PEG–SiO_2_ with PVA:PEG (90:10) with compositional mapping of O, Si, and C elements.

Bright-field (BF) TEM and high-angle annular dark-field scanning transmission electron microscopy (HAADF-STEM) imaging were performed to monitor the distribution of silica nanoparticles in the fiber core. As can be seen in Figure 5b,c, spherical nanoparticles of silica are distributed over the center of core fiber PVA–PEG–SiO_2_ with the dual polymer mixture PVA-PEG at a ratio of 70:30. The individual separated nanoparticles within the fiber are observed in the PVA–PEG–SiO_2_ (70:30) sample (Figure 5b,c), while they are mostly segregated in the sample with PVA-PEG mixed in a ratio of 90:10 (Figure 5a). The average particle size distribution of silica was calculated from STEM images, where the size distribution ranges from 20 to 120 nm, and mean diameter of silica *d* equals to 54 ± 17 nm. EDX compositional maps were registered for O, Si, and C elements to detect the distribution of silica spheres in the core fiber PVA–PEG–SiO_2_ (90:10) in Figure 5d.

The mixture of PVA to PEG with the ratio of 70:30 was selected as a working polymer composition of core for PVA–PEG–SiO_2_@PVA–GO core-shell fiber. The composite with ratios of polymers PVA:PEG (90:10) demonstrates agglomerates of silica that claim the feasibility of composite PVA:PEG (70:30), where PEG functions as a good dispergator. The increased PEG content prevents the distribution of silica nanoparticles without the agglomeration in the core fiber.

#### 3.2.4. Core-Shell Structure Confirmation by Fluorescent Microscopy

**Figure 6:**
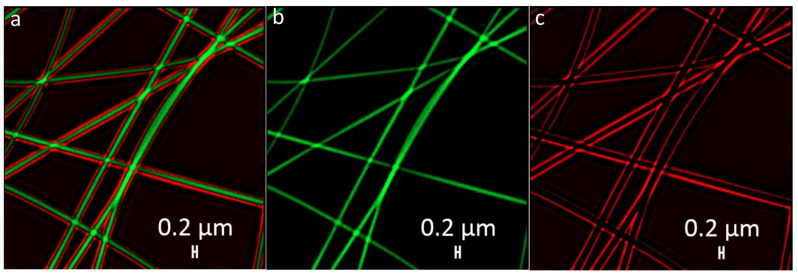
Representative fluorescence microscopy images collected from: (**a**) core-shell PVA–PEG–SiO_2_@PVA–GO fibers with the labelled bilayered structure; (**b**) FITC represents the core in green; (**c**) Rhodamine B indicates a shell of fiber colored in red.

The core-shell structures were labeled with FITC for the core and Rhodamine B for the shell, respectively. Figure 6 shows the bilayered structure including the core and shell colored with green and red, respectively. The calculated mean values for shell wall thickness and core were near 66 ± 18 nm and 173 ± 25 nm, respectively.

#### 3.2.5. Fiber Mat Degradation Analysis

To analyze the impact of GO, the experiment was carried out to determine the water uptake effect of PVA–GO composite with the use of SEM analysis of the fiber diameter after each step. The samples were stored in distilled water for 2 h during the experiment to study the fiber’s nearest degradation stage via SEM imaging. A PVA-based fiber mat was used as a control sample.

**Figure 7:**
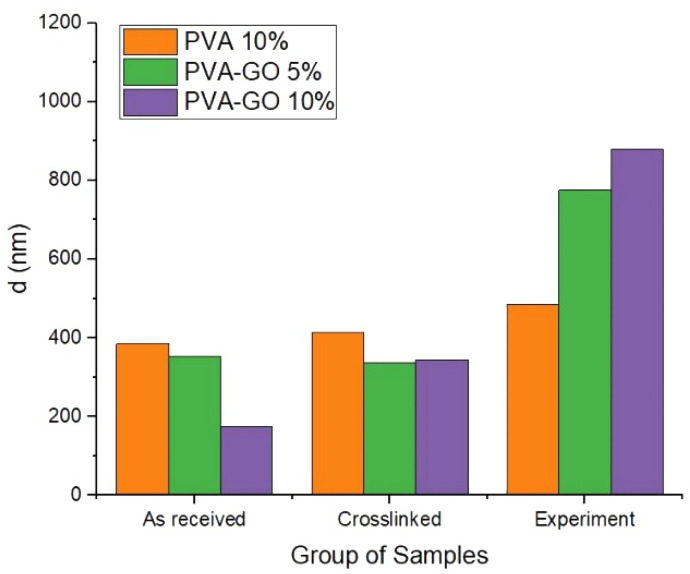
Size distribution of fibers as-received, crosslinked shell fiber mats, and after water immersion in 2 h.

Figure 7 demonstrates that fibers of PVA 10%, with the higher fraction PVA, show no significant swelling at the three stages. GO loading of 5 wt.% and 10 wt.% does not change the average fiber diameter significantly after methanol stabilization. However, the addition of graphene oxide up to 5 wt.% changed the swelling of fiber at least two times after being kept in water. The results show that more addition of GO (10 wt.%) resulted in the dramatic swelling of the PVA–GO composite after the water immersion and crosslinking. It demonstrates the high water uptake behavior for PVA–GO shell composite in the aqueous environment.

### 3.3. Wettability of Fiber Mats

The wettability of shell fiber and core-shell samples are assessed to trace the impact of GO and NPs of silica on the fiber composites (Table 3). The mean contact angle was calculated after the drop stabilized for 10 s before the drop was fully absorbed by the thin sample. The mean contact angle for PVA–GO 5% and PVA–GO 10% changes from 48 ± 7° to 39 ± 5° with the higher loading of GO fraction. The high hydrophilicity of graphene oxide results in the rapid absorption of fibers during the contact angle measurements. Being rearranged inside the fibers, GO nanoflakes form a high hydrophilic structured surface of PVA–GO composite due to their high hydrophilicity related to oxygen-containing functional groups of GO. The addition of GO rapidly increases the fiber diameter of PVA-based film, which increases the contact angle input for PVA–GO 5%. The contact angle for PVA–GO fiber mats with the growth of loading of GO to 10 wt.% is reduced due to the higher surface area characterized by the diminished diameter of fibers. After crosslinking, the contact angle was slightly changed for PVA–GO composites. Differently, it noticeably increased for the PVA 10% and core-shell PVA–PEG–SiO_2_@PVA–GO, where the shell contains 10 wt.% of GO loading. Then, the layered structure and bigger fiber diameter seem to decrease the wettability without leaving the hydrophilicity of the sample.

### 3.4. Spectroscopic Characterization

#### 3.4.1. Raman Spectroscopy of PVA–GO Shell and Core Composite

**Figure 8:**
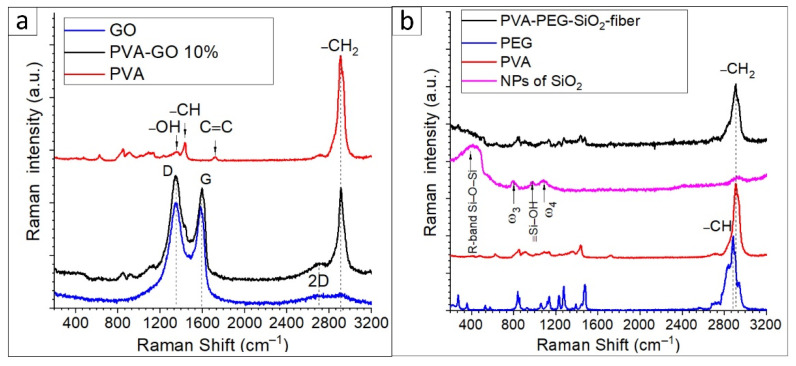
Raman spectra of samples: (**a**) GO and PVA–GO shell and PVA fiber mat; (**b**) PVA–PEG–SiO_2_ core fiber and the components (NPs of silica, PEG, and PVA).

The sample of as-received graphene oxide was monitored with Raman spectroscopy. The spectra of PVA–GO composites and GO were examined to uncover the characteristic D and G bands of graphene oxide, where the D band is in charge of the defects and vacancies of sp^2^ carbon hybridization, and the G band indicates the stretching C–C bonds. D (ca. 1353 cm^–1^) and G (ca. 1587 cm^–1^) bands indicated sp^2^ hybridization of the carbon atoms, while the following ratio of I_D_/I_G_ mainly defines the graphene oxide [32,39]. In Figure 8a, broad bands of doublets are located at ca. 2904 cm^–1^ and ca. 2700 cm^–1^ in the so-called 2D band. The shift of 2D bands defines the quality of stacking GO layers [39]. The found data in Figure 8a correspond to the reference [39], where the D and G bands’ centers peak at 1347.8 and 1583.07 cm^–1^ with the ratio of I_D_/I_G_ (1.02). The 2D band of the GO is at ca. 2692 cm^–1^, defining the GO layered structure. Due to the referenced data, the monolayered GO has a 2D band that occurs at 2679 cm^–1^; the shifted 2D band seems to indicate a multilayered structure of GO.

In Figure 8a, the spectra of PVA–GO present that the most prominent peak of PVA occurred at ca. 2905 cm^–1^ due to the –CH_2_ bond vibrations. For the pure PVA spectrum, the peaks at 1430 cm^–1^ and 1376 cm^–1^ are assigned to the wagging vibration of –CH and –OH in-plane vibration in the PVA molecules. The band at 1050–1100 cm^–1^ is attributed to C–O containing bond deformation vibration. The peaks at the regions of 1680–1720 cm^−1^ are related to the stretching of C=O bonds within the structure of PVA. The PVA–GO shell composite shows that the peaks of D and G occurred at 1347.8 and 1594.64 cm^–1^, respectively. The obtained peaks relate to the reported studies [40]. The presence of these three peaks in the Raman spectra of PVA–GO composites indicates the distribution of the GO flakes in the PVA–GO fibers. Additionally, a slight shift in 2D bands attributed to the GO is observed at ca. 2700 cm^–1^ for PVA–GO. The ratios of I_D_/I_G_ of the PVA–GO were calculated as 1.07, where the higher presence of covalent bonds formed in PVA–GO could cause the increase in the ratios I_D_/I_G_ [41].

Figure 8b demonstrates the spectra of PVA–PEG–SiO_2_ solution and contained additives. The spectrum of nanosilica shows the main features as the broad band centered at 416 cm^–1^ and peak at 799 cm^–1^, 976 cm^–1^, and 1087 cm^–1^ in Figure 8b. The band is located at the range 313–480 cm^–1^ and named R-band, indicating the Si–O–Si stretching vibration bonds. The width of this band is related to the dispersion of the Si–O–Si bond angle [42]. The asymmetric peak 799 cm^–1^ is considered as ω_3_ attributed to the random network of Si–O bonds, and the following peak at 976 cm^–1^ is attributed to the symmetric stretch vibrations of silanols in SiO_2_, ≡Si–OH groups [43,44]. The peak ω_4_ located in the frequency of 1087 cm^–1^ corresponds to the Si-O-Si bridging bond angle changes. Hence, the found frequencies of freeze-dried silica solution correlate with the referenced studies of nanosilica. Figure 8b shows the spectra of fiber mats (PVA–PEG–SiO_2_ and PVA 10 wt.%) and PEG powder as a component. The dual polymer composition is observed in the spectra of solutions and fibers. In Figure 8b, the fingerprints of PEG correspond to the core composite from the characteristic sequence of peaks in the range of 1500–200 cm^–1^ [45,46]. The high-intensive peak of the PEG spectrum located at 2887 cm^–1^ relates to the vibrations of symmetric C–H stretching bonds. In conclusion, the presence of NPs of silica can be monitored in the spectrum of the PVA–PEG–SiO_2_ fibers by the R-band.

#### 3.4.2. Fluorescence Spectroscopy Characterization of Crosslinked Core-Shell Fiber Mat

**Figure 9:**
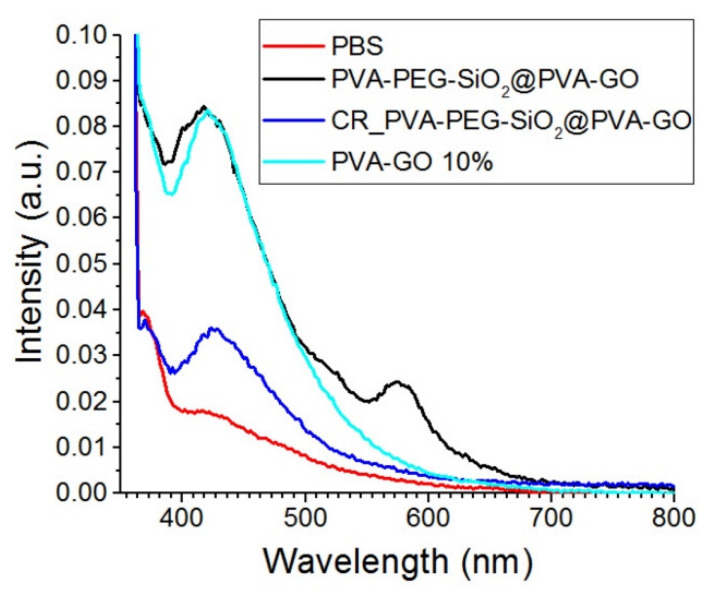
Fluorescence spectra of the crosslinked PVA–PEG–SiO_2_@PVA–GO core-shell composite and shell PVA–GO fibers excited at 330 nm.

The obtained PVA–PEG–SiO_2_@PVA–GO fiber mat was studied to trace its degradation in a medium close to the body conditions. The spectrum of PBS solution was estimated as the control in Figure 9. GO is pH-sensitive photoluminescent material and monitored with UV spectrophotometry. The spectrum of the GO is characterized by the prominent peaks occurring at the blue band (ca. 440 nm) and long-wavelength band (ca. 700 nm) in the solution pH = 2.5; however, the higher pH indicates the increase in the blue band emission [47]. In Figure 9, the blue band is merely observed for the GO contained in the crosslinked composite PVA–PEG–SiO_2_@PVA–GO. Due to the high pH, the presence of GO after the crosslinking with methanol was claimed by the blue band emission. After the excitation with the laser wavelength 330 nm, there are strong blue bands centered at 425 nm for all GO-based samples. Hence, the fluorescent emission shift of GO as it relates to factors such as the pH of the medium and the excitation wavelength should be considered. The consideration of these parameters aids in further study of the degradation behavior of GO-based composite.

In Figure 9, the secondary emission peak is observed for the spectra of non-crosslinked PVA–PEG–SiO_2_@PVA–GO core-shell. The peak (ca. 588 nm) of the non-crosslinked core-shell sample can be attributed to the oxidized debris potentially formed after the fiber degradation [48]. The dissolution of PVA and PEG increased the number of oxidized chemical bonds such as –COOH on the GO sheets, which affects the size of GO sheets. According to the mentioned above reference, the optical properties of oxidized debris in core-shell fibers are close to GO originated quantum dots, which can be further operated as nanofluorophores for spectroscopic imaging [22].

### 3.5. Mechanical Testing with DEBEN 200 N

**Figure 10:**
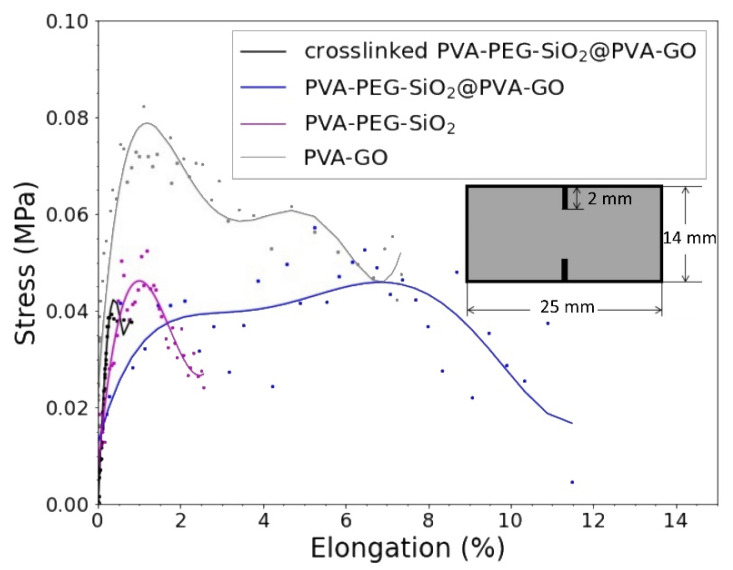
Stress-strain curves of PVA–PEG–SiO_2_@PVA–GO (core-shell), PVA–PEG–SiO_2_ (core), and PVA–GO (shell) fibers with the sample design for double edge notch test.

The stress-strain curves of tested samples are shown in Figure 10. The results of tensile measurements and elastic modulus of the core, shell, and core-shell fiber mats are included in Table 4. In this study, we tested the double notched samples cut from the composites: PVA–GO 10% as a shell, PVA–PEG–SiO_2_ as core, and PVA–PEG–SiO_2_@PVA–GO core-shell fiber mat. The tensile test of core composite (PVA–PEG–SiO_2_) demonstrates the reduced value of elongation at break in comparison with the PVA–GO shell. The presence of silica nanoparticles exerts the discontinuity in composite to cause faster failure at 2.57% of strain.

>However, the PVA–GO fiber mat shows an extended period of tensile deformation, where the fracture occurred at 6.28% of elongation. The core-shell fiber mat demonstrates the massive failure at 11.48% of strain.

The mechanical performance of core-shell fibers is complex, thus attributed to the input of core and shell parts. The increased resistance of tensile stress performs the synergetic impact of GO in the shell of core-shell composite PVA–PEG–SiO_2_@PVA–GO with the inner core diameter 173 ± 25 nm obtained from Section 3.2.4.

The stress-strain curve of the crosslinked PVA–PEG–SiO_2_@PVA–GO demonstrates the brittle behavior, where the break occurred at 0.8% of strain. The dimension of the fiber and crosslinked sample should be noted, where the bulkiest sample with the highest average fiber diameter performs the biggest value of elastic modulus of 9.47 MPa. The crosslinked samples require additional tests to verify and will be reported on in further study. The assigned crosslinking of PVA-based composite diminishes the free movement of polymer chains within the PVA network, reducing elasticity of fibers.

The PVA–GO composite serves as a protective shield for the core fiber and demonstrates higher mechanical properties as an elastic modulus. The strength of the core-shell structure with its potential drug loading capacity is speculated as a direction to the functionality of coaxial electrospinning [49,50,51,52].

## 4. Conclusions

In this work, we presented the detailed fabrication route of the multifunctional electrospun mats based on PVA polymer fibers with core-shell morphology. The fibers were modified with silica nanoparticles, PEG, and graphene oxide for the PVA–PEG–SiO_2_ core and PVA–GO shell fibers. The core composition of PVA:PEG with ratio 70:30 was suggested to suit the silica distribution within the PVA–PEG–SiO_2_ core without silica aggregation. The results of the study demonstrated that as-received solutions of GO and SiO_2_ mainly contain the solvents. This liquid part of the PVA ratio both affects the rheological properties of spinning solutions and results in the morphological features of fibers

Graphene oxide in the shell structure increases the mechanical strength and prolongs the elasticity of resulting fiber mats. GO concentration is a very important factor that regulates the fiber diameter and hydrophilicity of the mat. Fibers with GO content showed water uptake increased two times and decreased fiber diameter for content of GO (10 wt.%), relative to pristine PVA fibers.

The addition of silica solution and PEG significantly impacts such properties of solutions as conductivity and viscosity, which, finally, affects the quality of the fiber formation process and ultimate fiber diameter. Moreover, silica prevents swelling of the polymer network by reducing the movement of polymer chains and, consequently, inhibits the fiber swelling after crosslinking with methanol.

The core-shell structure with NP’s silica distribution was confirmed by TEM analysis and confocal fluorescent microscopy with the labeling agents of core-shell solutions. Imaging the separate core and shell structures with fluorescent dyes acquired evidence of the substance loading without the dilution within the fiber.

Core-shell fiber has less fiber diameter compared to the pristine PVA fiber and PVA–PEG–SiO_2_, but not compared to the PVA–GO 10%. Micromechanical testing unearthed the effect of GO additives on elasticity and tensile strength of the resulting fiber and claimed that the shell composite played the crucial role in the mechanical properties. The study with Raman spectroscopy of GO-based composite showed the changes in chemical structure for GO in terms of the layered organization and oxygen-containing groups of GO. The pH and time-dependent changeability of GO directly affects the results of electrospinning. PVA–PEG–SiO_2_@PVA–GO core-shell fibers showed good hydrophilicity, comparable with pristine PVA fiber, and fluorescence properties that have feasible application in the controlled drug molecules release [53].

This study demonstrates the prospects of the core-shell fiber mat additionally modified with the microrelief. The prepared fibrous mats possess the dual intercalation of substances into both shell and core. The obtained material is expected to solve the issue of the potential drug loading volume and thickness of film for wound healing applications.

## Figures and Tables

**Table 1:** The list of spinning solutions.

Name	Mass Ratio, wt.%				
	Liquid Part	PVA	PEG	SiO_2_	GO
PVA 10%	90.000	10.000	-	-	-
Core composite					
PVA-PEG	88.000	8.400	3.600	-	-
PVA–PEG–SiO_2_	87.995	8.400	3.600	0.005	-
Shell composite					
PVA–GO 10%	90.758	9.000	-	-	0.242
PVA–GO 5%	90.380	9.500	-	-	0.120

**Table 2:** The measurements of solutions.

Name	Mass Ratio of PVA, wt.%	Viscosity, mPa∙s	Shear Rate, s^–1^	Volume Electrical Conductivity, μS∙cm^–1^
PVA 10%	10	1058.9	3.1224	672
PVA-PEG	8.4	339.8	2.9594	538
PVA–PEG–SiO_2_	8.4	239.3	4.1350	1332
PVA–GO 5%	9.5	885.2	2.3752	662
PVA–GO 10%	9	452.2	2.2462	653

**Table 3:** The contact angle of PVA–GO 5%, PVA–GO 10%, and PVA–PEG–SiO_2_@PVA–GO before and after crosslinking.

Name of Sample	Average Diameter, nm	Contact Angle, °
As-received		
PVA 10%	386 ± 81	28 ± 3
PVA–GO 5%	354 ± 79	48 ± 7
PVA–GO 10%	174 ± 31	39 ± 5
PVA–PEG–SiO_2_@PVA–GO	261 ± 51	26 ± 3
Crosslinked		
PVA 10%	415 ± 88	37 ± 4
PVA–GO 5%	338 ± 47	50 ± 7
PVA–GO 10%	344 ± 114	39 ± 2
PVA–PEG–SiO_2_@PVA–GO	503 ± 137	34 ± 1

**Table 4:** The tensile properties of PVA–PEG–SiO_2_@PVA–GO, PVA–PEG–SiO_2_ (core), and PVA–GO (shell) fibers.

Name of Sample	Thickness, μm	Average Diameter, nm	Density, g∙cm^–3^	Elastic Modulus, MPa	Strength, MPa	Elongation at Break,%
shell PVA–GO 10%	50	174 ± 31	0.114 ± 0.020	8.40	0.0780	6.28
core PVA–PEG–SiO_2_	55	381 ± 131	0.147 ± 0.022	4.98	0.0474	2.57
core-shell PVA–PEG–SiO_2_@PVA–GO	32	261 ± 51	0.119 ± 0.007	3.17	0.0429	11.48
crosslinked core-shell PVA–PEG–SiO_2_@PVA–GO	121	503 ± 137	0.551 ± 0.078	9.47	0.0413	0.80

## Data Availability

Not applicable.

## Abbreviations

Polyvinyl alcohol (PVA); graphene oxide (GO); polyethylene glycol (PEG); tetraethyl orthosilicate (TEOS); phosphate buffered saline (PBS); fluorescein isothiocyanate (FITC); nanoparticles (NPs); FabLab and Machine Shop Shared Facility (FabLab); Scanning Electron Microscopy (SEM); Transmission Electron Microscopy (TEM); Bright-field (BF) TEM; High-angle Annular Dark-field Scanning Transmission Electron Microscopy (HAADF-STEM); energy-dispersive X-ray (EDX) analysis; Back-scattered Electron (BSE) detector; Digital Image Correlation (DIC).
